# Jacket Crowns
*Read before the Lake Erie Dental Society, May, 1903.


**Published:** 1904-06

**Authors:** A. C. M'Alpin

**Affiliations:** Warren, Pa.


### JACKET CROWNS.*

By Dr. A. C. M'Alpin, Warren, Pa.

The application of the jacket crown is indicated in those cases where the tooth is beyond successful structural restoration by filling, to peg teeth and to teeth that are slightly out of correct position and in those cases where the retention of the life of the teeth is desirable.

It is contended that a more intimate knowledge of and practice with this crown will induce many who now, with the utmost impunity, destroy tooth-life, to take the little extra pains necessary to produce the permanent artistic jacket, and at the same time conserve the normal life energy which is better, no matter what specious arguments are made to the contrary.

The jacket crown is simple in its construction and yet is susceptible to the greatest skill in its application. Briefly stated, the crown consists of a platinum shell fitted over a stub of tooth and upon which is fused porcelain body with a veneer facing.

As these crowns are used mostly on the anterior teeth I will describe a case as applied to an incisor. The tooth is dressed down to a wedge-shaped stub with lingual ground sufficiently to accommodate a 28-gauge plate and a labial space of at least 16-gauge. The cervical ridge is obliterated.

A cylindrical platinum band lap joint 30-gauge as long as the original crown is fitted at the cervix. This is then held in position while on the lingual portion a line is scratched along the cervical margin and at the linguo-distal and mesial angles. The space within these lines is then ground with a coarse dry stone until very thin and the metal burnished down until the lingual aspect is shaped as the surface on the finished crown should appear. This surface is then reinforced by another platinum plate, 30-gauge, which is burnished and shaped to it and soldered with pure gold. The labial portion of the cylinder is then ground in the same way and the metal adapted to the stub by rough-end instruments to prevent burnishing as the fresh-ground surface affords strong adhesion to the porcelain body.

High heat porcelain body is then fused to the ground labial surface, bringing the body to a good flux only. Then a suitable veneer facing ground thin is attached with added body by the second baking. The metal back is finished as metal crowns usually are and the jacket is adjusted as is the ordinary shell crown.

In this era of porcelain dental work the jacket fills a position which is unassailable by any other form of crown. Attempts are made to attach ordinary facings to gold shells but these must necessarily be bulky and bungling when compared to the true jacket crown.

Once one familiarizes himself with the possibilities in the application of the jacket crown, he opens the way to far more satisfactory work than was possible before.

It is worth one's while to begin porcelain

---

*Read before the Lake Erie Dental Society, May, 1903.

work as early as possible for its varied uses tend to bring out the operator's talent, manipulative and artistic ideas, more than in any other phase of dental labor.

